# Chronic Co-administration of Methylphenidate and Fluoxetine Reduces Striatal NMDA Receptor Binding in Adolescent Rats

**DOI:** 10.1007/s11064-026-04743-5

**Published:** 2026-04-03

**Authors:** George Lagamjis, Divon Slayton, Huy Lu, Caleigh Hoerner, Abigail M. Lantry, Matthew Marion, Dan Elman, Michael Hadjiargyrou, David Komatsu, Panayotis K. Thanos

**Affiliations:** 1 Behavioral Neuropharmacology and Neuroimaging Laboratory on Addictions, Clinical Research Institute on Addictions, Department of Pharmacology and Toxicology, Jacobs School of Medicine and Biomedical Sciences, University at Buffalo, 1021 Main Street, Buffalo, NY 14203-1016 USA; 2 D’Amore-McKim School of Business, Northeastern University, Boston, MA USA; 3 Department of Psychiatry, Cambridge Health Alliance, Harvard Medical School, Boston, MA USA; 4 Department of Biological and Chemical Sciences, New York Institute of Technology, Westbury, NY 11568 USA; 5 Department of Orthopaedics and Rehabilitation, Stony Brook University, Stony Brook, NY 11794 USA; 6 Department of Psychology, State University of New York at Buffalo, Buffalo, NY USA; 7 Department of Exercise and Nutrition Sciences, State University of New York at Buffalo, Buffalo, NY USA

**Keywords:** Methylphenidate, Fluoxetine, NMDA, Adolescence, Addiction

## Abstract

Methylphenidate (MP), a widely used medicine for attention deficit/hyperactivity disorder (ADHD), is commonly prescribed in combination with selective serotonin reuptake inhibitors (SSRI) e.g., fluoxetine (FLX). However, neurochemical effects of the MP + FLX combination have not been sufficiently elucidated. Given the pivotal role played by glutamatergic signaling in psychostimulant responses and corticostriatal plasticity, we employed a 2-level factorial design to assess how the individual treatments and their co-administration affect N-methyl-D-aspartate receptor (NMDAR) binding using [³H] MK-801 in vitro autoradiography. Three-week-old male rats were randomized into four groups: MP (30/60 mg/kg), FLX (20 mg/kg), MP + FLX (30/60 mg/kg and 20 mg/kg), and vehicle. Treatment was administered for four weeks using a dual bottle drinking paradigm that models human dosing and pharmacokinetics. Following the treatment, [³H] MK-801 in vitro autoradiography was performed on coronal brain sections. The MP + FLX group significantly decreased NMDA binding levels in the dorsal caudate-putamen (DCPU) (39%), ventral caudate-putamen (VCPU) (36%), and nucleus accumbens (Nac) (34%), compared to vehicle. Rats in the MP group demonstrated reduced NMDA binding in the DCPU only, and there were no significant differences in binding for the FLX group. These findings indicate that MP + FLX coadministration yields a reduction in NMDAR binding across the striatum, an effect not produced by either of the drugs. Thus, reduced NMDA binding following MP + FLX treatment may contribute to dysregulations in memory, motor, and reward systems. Further studies are warranted to evaluate the neurochemical, neurodevelopmental, and clinical correlates of MP + FLX glutamatergic effects in adolescent patients.

**Supplementary Information:**

The online version contains supplementary material available at 10.1007/s11064-026-04743-5.

## Introduction

Methylphenidate (MP) is the most commonly prescribed psychostimulant for attention-deficit/hyperactivity disorder (ADHD) [1, 2], a condition whose prevalence continues to rise among adolescents [1]. ADHD has been linked to dysregulated dopaminergic frontostriatal pathways [2], and modulation of these pathways contributes to the therapeutic effects of MP [3]. MP effectively reduces hyperactivity, impulsivity, and inattention [4, 5] through inhibition of both the dopamine transporter (DAT) and norepinephrine transporter (NET), increasing synaptic dopamine (DA) and norepinephrine (NE), thereby enhancing prefrontal cognitive function [6, 7]. Although well tolerated [5], MP use is associated with adverse effects, including anxiety, insomnia, and transient skeletal bone deficits [8–13]. It is also known that MP carries a high potential for misuse due to its affinity for dopamine [14, 15]. Chronic MP exposure also produces wide-ranging neurobiological and developmental consequences, including effects on bone health [9–12], neurochemistry [16–21], brain gene expression [22], brain glucose metabolism [16, 23], and overall physiology [24–27]. With low doses, MP has been seen to increase NMDAR expression, whereas high doses decrease expression [28]. Previously, we investigated the effects of chronic oral MP use on NMDARs, and following a 3-month treatment, and subsequent in vitro autoradiography analysis, we revealed dose-dependent effects on glutamate signaling [18].

It is also broadly established that over 60% of individuals with ADHD have a comorbid physical, mental, or developmental disorder [29–32]. As major depressive disorder (MDD) becomes increasingly prevalent [33], and selective serotonin reuptake inhibitors (SSRIs) such as fluoxetine (FLX) are often prescribed concurrently with MP. FLX acts primarily by blocking the serotonin transporter (SERT), thereby increasing synaptic serotonin (5-HT) [34], but its neurochemical effects extend beyond serotonergic signaling. Increasing extracellular 5-HT can drive co-release of 5-HT and DA through DAT-mediated uptake [35]. Further, FLX can alter glutamate neurotransmission [36, 37], with studies reporting decreased glutamate levels throughout the brain [38, 39]. Despite the frequency with which MP and SSRIs are co-prescribed in clinical practice, the extent of the neurobiological consequences of their combined use remains to be determined.

Evidence from preclinical studies reveals that FLX potentiates MP-induced gene regulation when delivered via either intraperitoneal (IP) injections or oral administration [40–42]. Combined MP-FLX treatment also produces neuronal [41–43] and behavioral [24, 41, 42, 44, 45] effects not observed with either drug alone, including enhanced anxiolytic and antidepressant-like behaviors [26, 44], reductions in striatal DA type-2 receptor (D2) expression [46], alterations in cannabinoid receptor density [47], and in contrast to MP monotherapy [48], enhanced drug-seeking behaviors [49]. Developmentally, MP-FLX co-exposure significantly impairs weight gain [13, 25] and influences growth plate morphology [13, 25]. These findings indicate that MP and FLX interact across multiple neurochemical and physiological systems, suggesting synergistic rather than purely additive effects. Such interactions provide a strong rationale for the use of a two-level factorial design to elucidate the independent and combined drug effects.

Glutamate, acting through NMDARs, is the major mediator of excitatory neurotransmission [50] and is essential for synaptic plasticity, cognitive processing, and corticostriatal function [51–53]. Dysregulated glutamate signaling is implicated in a range of neuropsychiatric and neurodegenerative disorders, including depression [54–57], Parkinson’s disease [58], Alzheimer’s disease [59], and substance use disorder [60, 61]. DA and glutamate systems interact extensively within the striatum [62, 63], and psychostimulant exposure engages both pathways [60, 64, 65]. Previous literature showing that altering NMDAR function during adolescence results in long-term consequences, as described above [54–57, 66–68], no studies have examined how MP and FLX together affect NMDAR binding in the adolescent brain. This represents a critical gap, particularly given the clinical prevalence of co-prescribing and the importance of glutamatergic signaling.

To address this gap, we used an oral, voluntary, limited-access drinking paradigm that models human pharmacokinetics [24, 45] to administer MP, FLX, or their combination for four weeks during adolescence. Using this methodology, we have previously shown that chronic oral MP treatment alone has distinct effects on dopaminergic signaling [effects on DA transporters (DAT) and DA Type-1 receptors (D1R)] [20]. Further, we have previously shown that FLX potentiates MP-induced gene regulation [42], decreases D2 receptor levels in the striatum [46], increases the cannabinoid receptor type-1 expression in the somatosensory forelimb region [47], and increases microglial activation [69]. Moreover, our lab has previously shown that combined MP and FLX enhance drug-seeking behaviors [49] and induce a decrease in anxiety and depressive-like behaviors greater than either drug alone [44, 49]. Our prior work on MP and NMDA has shown that chronic oral MP treatment has dose-dependent effects on NMDAR binding [18]. The present project builds on this foundation to investigate whether combined MP + FLX treatment produces greater reductions in NMDAR binding than either drug alone using in-vitro autoradiography in order to assess localization of receptors in specific regions of the brain. In-vitro autoradiography allows us the ability to quantify NMDAR binding in specific brain regions and compare receptor density across treatment groups. We hypothesized that MP + FLX co-administration would produce reductions in NMDAR binding across striatal and cortical regions.

## Methods

### Procedure

**Fig. 1:**
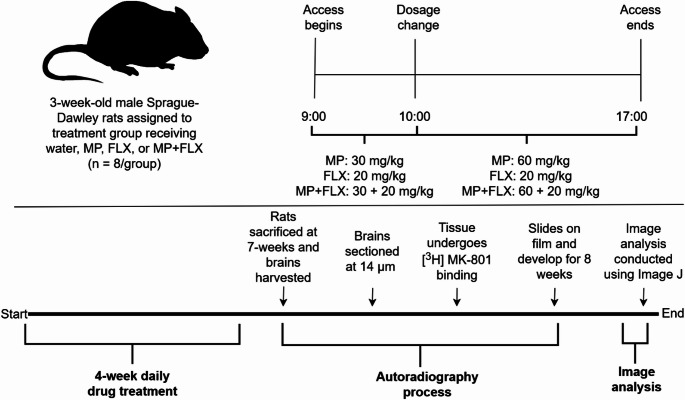
Timeline for the dosing schedule for animals from 9:00–17:00. From 9:00–10:00, rats in the MP group received a dosage of 30 mg/kg, rats in the FLX group received a dosage of 20 mg/kg, and rats in the MP + FLX group received a dosage of 30 mg/kg MP and 20 mg/kg FLX. From 10:00–17:00, rats in the MP group received a dosage of 60 mg/kg, rats in the FLX group received a dosage of 20 mg/kg, and rats in the MP + FLX group received a dosage of 30 mg/kg of MP and 20 mg/kg of FLX

Three-week-old Sprague Dawley rats were housed individually in humidity-controlled rooms (22 ± 2 ◦C, 50 ± 10% relative humidity) with a reverse light-dark cycle (lights off at 8:00 h). Rats were randomized by into four different treatment groups (*n* = 8/group) by body weight. Groups consisted of Control (Water), MP, FLX, and MP + FLX. MP hydrochloride was dissolved in distilled H_2_O (dH_2_O) to produce 30 and 60 mg/kg solutions. FLX was dissolved in dH_2_O to produce 20 mg/kg solutions (Sigma Aldrich, St. Louis, MO). Bottles were made fresh daily. Solutions were based on body weight and average fluid consumed from the previous three days. Treatments were administered every day for four weeks using a previously established dual-bottle drinking paradigm over the course of 8 h [45]. Specifically, the dual-bottle drinking paradigm is organized around rats’ typical drinking behavior, with treatment administered for one hour in the morning (9:00–10:00) and then for seven hours for the rest of the day (10:00–17:00), as previously described [45] (Fig. 1). Because rats consume larger volumes of liquid after overnight fluid restriction, the dose in the first bottle given in the morning is lower than the one in the afternoon. During the first hour, rats in the MP group receive a dosage of 30 mg/kg, rats in the FLX group receive a dosage of 20 mg/kg, and rats in the MP + FLX group receive a dosage of 30 mg/kg MP and 20 mg/kg FLX. During the following seven hours, rats in the MP group receive a dosage of 60 mg/kg, rats in the FLX group receive a dosage of 20 mg/kg, and rats in the MP + FLX group receive a dosage of 60 mg/kg MP and 20 mg/kg FLX. Note that the dosage of FLX is the same in both bottles due to previous experiments conducted by our lab that show that this dose is clinically relevant and is sufficient to potentiate the effects of MP [42]. This dual-bottle paradigm was utilized because it achieves the target plasma concentration range seen clinically [45].

At the end of the treatment period, rats were euthanized with isoflurane (3.0%) and brains were isolated, flash frozen, sectioned at 14 mm and subsequently stored at −80 °C. All experiments were approved by the University at Buffalo Institutional Animal Care and Use Committee.

### NMDAR Autoradiography

NMDAR binding was measured using [³H] MK-801in vitro autoradiography and was performed as previously described [70]. Slides were preincubated for 30 min at room temperature (RT) in a 50 mM Tris Acetate buffer (pH = 7.4). After, 30 µM glutamate, 10 µM glycine, and 4.2 nM [³H] MK-801 (specific activity = 26.4 Ci/mmol) were all added to the preincubation buffer. Slides incubated in this solution for 4 h at RT. Non-specific binding was determined in the presence of 100 µM unlabeled MK-801. Afterwards, slides were dipped in preincubation buffer at 4 °C, and incubated in preincubation buffer for an additional 90 min, followed by a briefly immersion in dH_2_O at 4 °C.

Slides were opposed to BioMaxXAR film for 8 weeks. [³H] MK-801 binding density was calculated using ImageJ software with two independent individuals performing the analysis of each film. Each individual was blinded to the other’s data, and no significant difference between each individual’s measurements was found. Measurements of the ROIs were carried out at the bregma coordinates taken from the Paxinos and Watson rat brain atlas [71]. ROIs analyzed included prelimbic (PrL), infralimbic (IL), secondary motor (M2), primary motor (M1), cingulate (Cg), insular (Ins), nucleus accumbens (Nac) (separated into the nucleus accumbens core (Nac Core) and nucleus accumbens shell (Nac Shell)), somatosensory jaw (S(Jaw)), somatosensory forelimb (S(FL)), somatosensory upper lip (S(ULP)), somatosensory hindlimb (S(HL)), somatosensory barrel field (S(BF)), somatosensory trunk (S(Tr)), primary somatosensory (S1), secondary somatosensory (S2), caudate Putamen (CPU) (separated into the dorsal caudate putamen (DCPU), ventral caudate putamen (VCPU), dorsomedial caudate putamen (DMCPU), dorsolateral caudate putamen (DLCPU), ventromedial caudate putamen (VMCPU), and ventrolateral caudate putamen (VLCPU). (Note that the VCPU and DCPU are measurements taken of the CPU in the posterior of the brain, where they are no longer separated into quadrants.), piriform cortex (Piri), globus pallidus (GP), hypothalamus (Hyp), thalamus (Th), entorhinal cortex (Ent), ectorhinal cortex (Ect), perirhinal cortex (PRh), amygdala (Amy), hippocampus (HP), retrosplenial (Rs), auditory cortex (Aud), visual cortex (Vis), substantia nigra (SNR), colliculi (Colli), and the cerebellum (CB). A total of 32 brains were analyzed, with an estimated yield of 40 sections per brain. Regions of interest are matched from the brain slide to from Paxinos and Watson Brain Atlas, 6th edition [71]. Specific binding was calculated by subtracting the nonspecific binding from the total binding and is expressed in µCi/g.

### Statistics

Specific [³H] MK-801 binding for each ROI was analyzed using one-way ANOVA. All statistical analyses and graphing were performed using GraphPad Prism 8, with statistical significance set at α = 0.05. As previously stated, two individuals completed the ROI and statistical analysis completely independent of each other. A paired t-test was performed against the two sets of data and showed no significant difference between either individual’s ROI analysis. Subsequently, both sets of data were averaged together to perform the statistical analysis mentioned above. Certain ROIs were combined to investigate the significance in the entire brain region as opposed to segmented regions. In this study, we combined the DCPU, DMCPU, and DLCPU to form the Dorsal CPU (DCPU), the VCPU, VMCPU, and VLCPU to form the Ventral CPU (VCPU), and the Nac Core and Nac Shell to form the Nucleus Accumbens (Nac). Other ROIs with similar functionality were also combined, such as the M1 and M2, S1F and S1H, and S1 and S2 which yielded no significant differences. All data were tested for normality before determining significance. Post-hoc Tukey’s honestly significant difference (HSD) tests were performed to identify differences for all significant main effects. Values are expressed as a total [³H] MK-801 binding means (µCi/g) ± SEM for NMDARs.

## Results

**Fig. 2:**
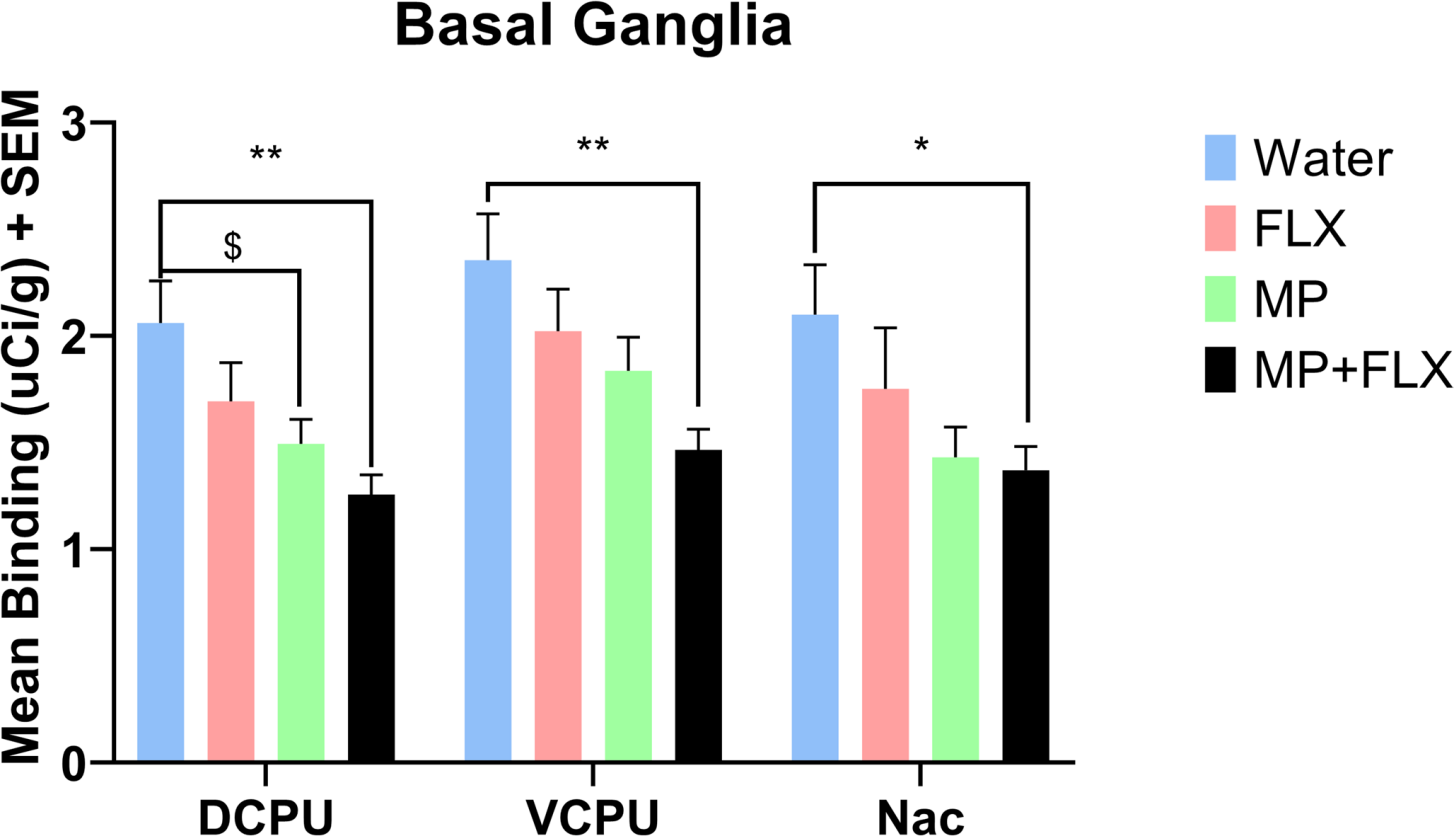
Mean [³H] MK-801 binding (µCi/g) ± SEM in the Basal Ganglia, specifically the Dorsal Caudate Putamen (DCPU), Ventral Caudate Putamen (VCPU), and Nucleus Accumbens (Nac), following 4 weeks of treatment, with *n* = 8 per group. * denotes a significant decrease (*p* < 0.05) between MP + FLX and Water. ** denotes a greater significant decrease (*p* < 0.01) between MP + FLX and Water. $ denotes a significant decrease (*p* < 0.05) between MP and Water. Results from post-hoc Tukey’s multiple comparisons test

**Fig. 3:**
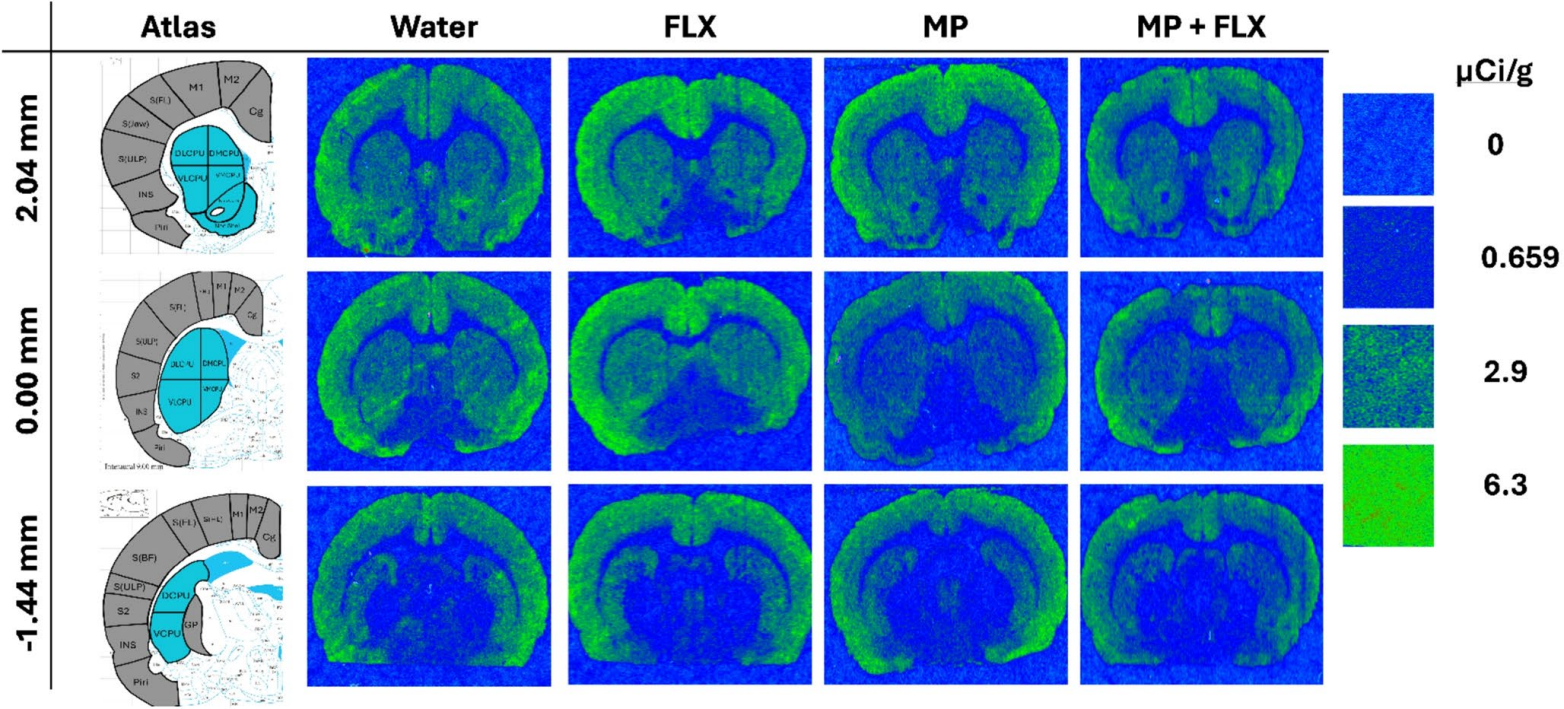
Representative figures showing NMDAR binding using [³H] MK-801 to assess binding levels, with included atlas figure showing: Cingulate (Cg), Primary Motor (M1), Secondary Motor (M2), Somatosensory Hindlimb Region (S(HL)), Somatosensory Forelimb Region (S(FL)), Somatosensory Upper Lip (S(ULP)), Somatosensory Jaw Region (S(Jaw)), Somatosensory Barrel Field Region (S(BF)), Secondary Somatosensory (S2), Insular (Ins), Dorsomedial Caudate Putamen (DMCPU), Dorsolateral Caudate Putamen (DLCPU), Dorsal CPU (DCPU), Ventromedial Caudate Putamen (VMCPU), Ventrolateral Caudate Putamen (VLCPU), Ventral Caudate Putamen (VCPU), Nucleus Accumbens Core (Nac Core), Nucleus Accumbens Shell (Nac Shell). Regions of interest are taken from Paxinos and Watson Brain Atlas, 6th edition [71]. Areas shaded blue in the atlas indicate regions of statistical significance. Areas shaded with gray indicate regions with no significant difference between treatment groups

A series of one-way ANOVA analyses was performed on [³H] MK-801 binding for each region of interest. A significant decrease in [³H] MK-801 binding was observed in the MP + FLX group as compared to the Water control group. Specifically, statistically significant decreases in [³H] MK-801 binding was observed in the DCPU [F (3, 92) = 4.956; *p* = 0.0019], the VCPU [F (3, 92) = 4.479; *p* = 0.0026], and the Nac [F (3, 57) = 3.040; *p* = 0.0454] (Figs. 2 and 3).

**Fig. 4:**
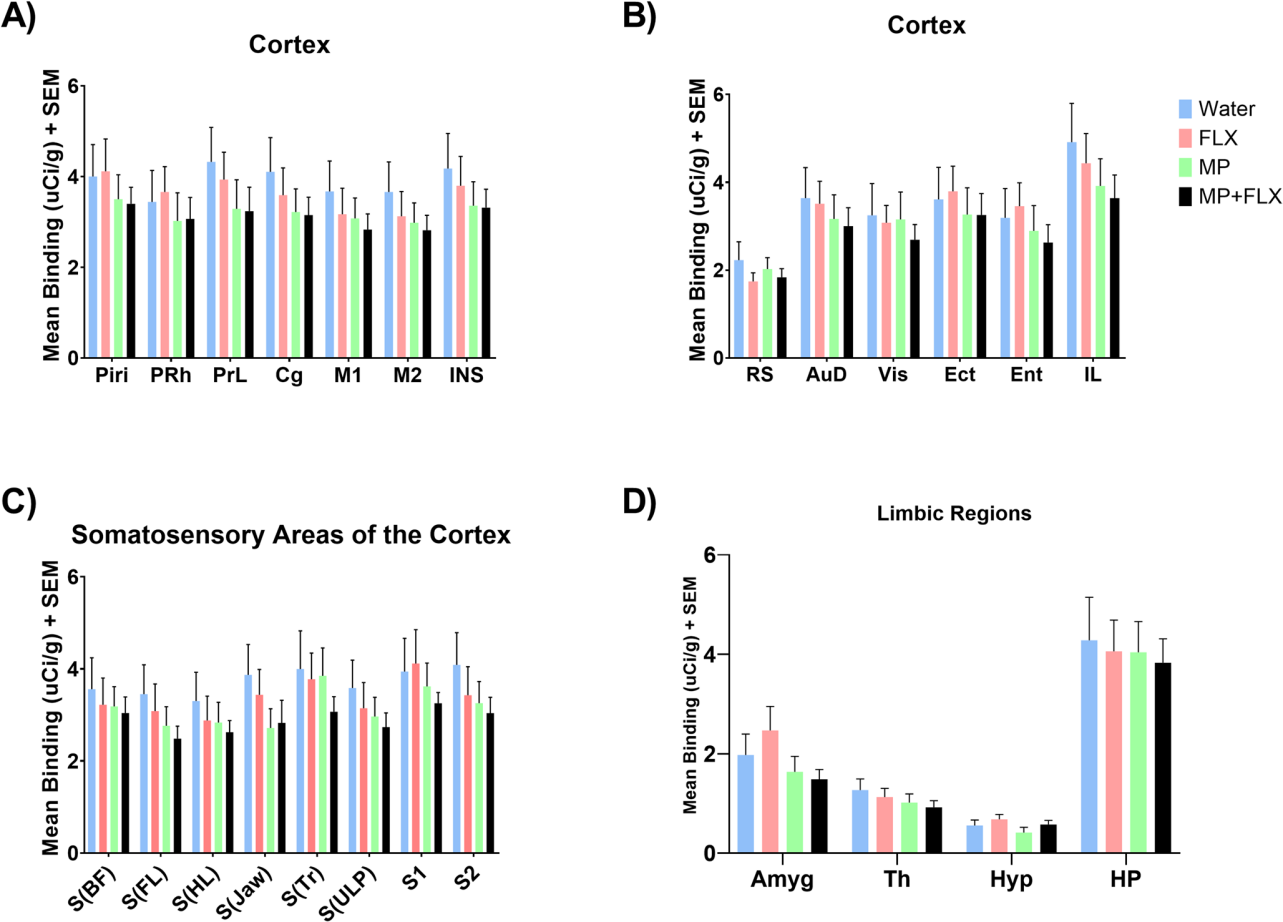
Mean [³H] MK-801 binding (µCi/g) ± SEM in the Basal Ganglia following 4 weeks of treatment, with *n* = 8 per group. No significant difference (*p* > 0.05) was observed across any of the groups in any of the ROIs. **A** Represents regions of the cortex. Regions include the Piriform Cortex (Piri), Perirhinal Cortex (PRh), Prelimbic (PrL), Cingulate (Cg), Primary Motor (M1), Secondary Motor (M2), and Insular (Ins). **B** Represents additional regions of the cortex. Regions include the Retrosplenial (Rs), Auditory Cortex (Aud), Visual Cortex (Vis), Ectorhinal Cortex (Ect), Entorhinal Cortex (Ent), and Infralimbic (IL). **C** Represents the somatosensory regions of the cortex. Regions of interest include the Somatosensory Barrel Field Region (S(BF)), Somatosensory Forelimb Region (S(FL)), Somatosensory Hindlimb Region (S(HL)), Somatosensory Jaw Region (S(Jaw)), Somatosensory Trunk Region (S(Tr)), Somatosensory Upper lip Region (S(ULP)), Primary Somatosensory (S1), and Secondary Somatosensory (S2). **D** Represents the limbic regions of the brain. These regions include the Amygdala (Amyg), Thalamus (Th) Hypothalamus (Hyp), and Hippocampus (HP). Results from post-hoc Tukey’s multiple comparisons test

In addition, the MP-treated rats were shown to have statistically significant decreases in [³H] MK-801 binding in the DCPU [F (3, 92) = 4.956; *p* = 0.0493] as compared to the Water control group. No significance was observed across all other brain regions when comparing the MP treated rats to controls (Supp. Table 1, Fig. 4).

## Discussion

This study investigated the effects of combined chronic MP and FLX treatment on NMDAR binding. A limited-access dual-bottle voluntary drinking paradigm was employed, which has been demonstrated to deliver a pharmacokinetic profile seen in clinical MP and FLX use [45]. Following 4 weeks of treatment, rats in the MP + FLX group showed significantly decreased NMDAR binding in the DCPU, VCPU, and Nac compared to the control group, which received water. Additionally, in the MP group, we observed a significant decrease in the DCPU compared to the control. However, there were no significant differences between the other ROIs.

Changes in NMDAR binding can influence memory formation, long-term potentiation & depression, and synaptic plasticity [51–53]. NMDARs contribute to the development and refinement of functional synapses [72–74]. Adolescence marks a period during which balanced excitation and inhibition is established [72], and aberrations have been linked to various conditions across different brain regions. We observed a decrease in NMDAR binding within the DCPU, and previous work has implicated altered NMDAR function in neurologic disorders such as Alzheimer’s [59] and Parkinson’s [58]. Further, given that the DCPU and VCPU are enriched in NMDARs, we hypothesize that the observed reduction in binding contributes to a dysregulation in reward processing [75]. In animal models, altered NMDAR function within the CPU has been linked to drug sensitization, drug-seeking behaviors, and cue association [18, 60]. One theory is that impaired NMDAR function may promote drug-seeking behaviors by decreasing inhibitory control on the DA system [76, 77]. Our laboratory previously showed that while MP altered NMDAR binding levels, the effects were reversible following abstinence [18]. However, FLX has been shown to potentiate MP-induced gene regulation in the striatum and thus, a similar abstinence period (1 month) may elicit different results.

MP has been shown to have dose-dependent effects on the glutamatergic system; however, these effects are often inconsistent [28, 78]. Urban et al. (2013) found a reduction in NMDAR expression in the prefrontal cortex following a single IP injection of MP (1 mg/kg), and, in contrast, Cheng et al. (2014) reported increased surface expression following low-dose IP injections (0.5 mg/kg). Further, higher doses (10 mg/kg) were shown to decrease NMDAR expression in the prefrontal cortex [28]. We observed no significant differences in binding in any cortical regions, and this difference may be due to our study, which utilized an oral-dosing paradigm, while the aforementioned studies used IP injections. IP injections of as low as 0.5 mg/kg MP elicit result in plasma concentrations at the maximum clinal dose within minutes, whereas oral dosing peaks are reached hours after administration [79]. This suggests that IP injections greater than this would result in plasma concentrations exceeding clinical doses, whereas our model has been shown to elicit a pharmacokinetic profile mimicking clinical use [45]. Additionally, these studies used western blotting, whereas our present study used in vitro autoradiography, which allows for quantifying NMDARs in distinct neuroanatomical regions. Furthermore, our voluntary drinking paradigm minimizes stress, so the observed results are likely the result of drug treatment in the absence of stress. We also observed no significant differences with low-dose MP but significant reductions in NMDAR binding in the PrL region at high doses [18]. In contrast, in the present study, we did not observe any significant reductions in any cortical regions. While we employed the same dual-bottle paradigm, our previous study assessed binding after 13 weeks, while the present study examined effects after 4 weeks. Further, our previous study used two doses of MP, whereas in the present study, we only administered a single dose. Consistently, we observed a significant reduction in the CPU and Nac. Together, these results support the notion that, following high doses of MP, subsequent decreases in NMDAR binding are observed [28, 80]. Length of treatment could be a possible explanation for the difference in these regions.

There is evidence implicating NMDAR dysfunction in the pathophysiology of depression [54–57], specifically overactivation [81]. NMDARs can be inhibited by FLX [82, 83]. It was also shown that 5-HT modulation plays a role in regulating NMDARs [84]. Previously, we also demonstrated that chronic MP treatment alone alters NMDAR binding in a dose-dependent manner [18], and the present study extends this work by testing not only MP at clinically relevant high doses, but also FLX alone and in combination with MP. We found significant decreases in NMDAR binding in the combined treatment group, suggesting that co-administration produces synergistic effects that are greater than those of either drug alone. This is also consistent with our previous findings of FLX potentiating MP-induced gene regulation and behavioral changes [42, 44]. In contrast, Vilazodone, a novel SSRI, has been shown to have no potentiating effects on MP-induced neuropeptide expression [85], suggesting that the observed interactions between MP and FLX may not extend to other SSRIs, thus presenting a potential alternative treatment without these potentially negative effects.

The DCPU consists of the DLCPU and DMCPU, key structures in the nigrostriatal pathway [86]. The DCPU is involved in associative learning [87], motor functioning [88], addiction, and reward processing [89]. Dopaminergic (DAergic) neurons project into the CPU, where they stimulate medium spiny neurons (MSN) expressing either D1Rs mediating the direct pathway, or D2Rs mediating the indirect pathway [90]. NMDARs are highly populated on MSN, along with DA receptors [62], and DA was demonstrated to modulate NMDAR responses [63]. Overactivity in the indirect pathway was documented to have a role in the onset of Parkinson’s disease, characterized by the degeneration of DAergic neurons, which increases the inhibition of movement [58, 91, 92], with greater dysfunction observed in the DCPU [93]. The mechanism underlying this is unknown; however, the relationship between DA and NMDA may be involved. Atypical NMDAR function may play a role, as increased NMDAR binding has been observed in both human tissue and Parkinsonian animal models [94, 95], and NMDAR antagonists have been shown to reduce symptoms [96]. Downregulation may play a role in mediating reduced binding, as it has been shown that NMDARs can downregulate in response to overactivation [97, 98]. Additionally, DA modulation may be involved, as there are known interactions between NMDARs and DA receptors that modulate NMDAR activity [63]. We hypothesize that this may be mediated, in part, by D2Rs, as we previously observed combined MP and FLX to decrease D2R binding in the striatum [46, 49].

The DLCPU is essential for habitual behavioral responses [99, 100]. Goodman et al. (2023) found that compared rats receiving a single injection, rats receiving repeated injections of an NMDA antagonist into the DLCPU were unable to consolidate memory. In contrast, the single-treatment group showed no impairment in memory retrieval [101]. Further, the DMCPU was demonstrated as being essential for goal-directed behaviors [102], with lesions shown to impair the acquisition of goal-directed actions [102]. This suggests that dysfunctioning NMDARs may result in impaired memory for both habitual and goal-directed behaviors, depending on the region affected.

The VCPU is associated with reward-processing, cognition, and motor function [103, 104], and with connections to the limbic system [105]. Decreased caudate volume was seen in the VCPU in later stages of Huntington’s disease; however, it is typically unaffected [106]. Compared to the DCPU, VCPU dysregulation has been primarily associated with mood disorders [103]. For example, dysregulation of the VCPU was observed in binge eating disorder [107], anorexia nervosa [108], bipolar disorder [109], depression, and obsessive-compulsive disorder (OCD) [110]. Further, the VCPU was observed to have a distinct functional connectivity involved in affective functioning, such as pain perception and fatigue [103]. Additionally, the loss of NMDAR on DAergic neurons was shown to induce symptoms associated with mood disorders [111]. Deep brain stimulation was shown to attenuate NMDAR functioning in the presence of NMDAR antagonists [112, 113] and alleviate psychiatric symptoms [103, 110, 114]. These results suggest that altered NMDAR activity in the VCPU may contribute to the development or exacerbation of mood disorders.

The ventral striatum consists of the olfactory tubercle and Nac, which is further divided into the core and shell [115, 116]. The ventral striatum receives DAergic inputs from the ventral tegmental area (VTA) [117, 118], and is responsible for the establishment and preservation of addiction [14, 15]. The VTA was shown to play a role in motivation, cognition, and reward processing [14, 119]. The shell is critical for reward-processing, motivation, and reinforcing behaviors [120, 121]. Similarly, the core is associated with reward processing, acting as a mediator and presenting learned cue associations to the shell, while also having a role in the reward-related motor function [120–122]. The NMDAR GluN1 and GluN2 subunits are necessary for receptor binding and function [123, 124]. Knockout of the GluN1 subunit in both direct and indirect MSN inhibits the induction of cocaine-induced conditioned place preference [125]; however, there is evidence that this does not occur when isolated to one type of MSN [60, 61]. Expression of GluN2B has been positively correlated with drug sensitization in the Nac [60, 64, 65]—and expression of GluN2B was observed to decrease in chronic methamphetamine use [64]. Thus, we hypothesize that our observed decreased binding in the Nac may be a result of compensatory effects mediated by NMDAR subunit expression.

Our findings have shown that the combined effects of chronic MP and FLX treatment surpass the effects of either drug alone, not just affecting the DAergic system, but also the glutamatergic system. Evidence supports the importance of glutamatergic signaling in the striatum, and altered striatal NMDAR function may lead to dysfunction in memory formation and retrieval, motor functioning, and increased susceptibility to addiction. These results serve to inform us about the neuropharmacological interactions between drug treatments and represent significant data, especially for individuals who both clinically and illicitly use MP in combination with FLX.

### Limitations and Future Directions

We have reported sex-dependent dimorphic effects with MP [43]; however, we have not identified any sex-dependent interactions with combined MP and FLX use, and as such, future studies should include both males and females. Additionally, our study only assessed treatment after 4 weeks; future research should assess longer durations as well as assess multiple time points throughout development. Further, with emerging evidence suggesting these potentiating effects are not observed with novel SSRIs, future studies should consider alternative combined treatments. Lastly, additional research is necessary to investigate the effects of combined MP and FLX treatment on NMDAR-mediated neurotransmission.

## Conclusion

The present study assessed the effects of both individual and combined chronic MP and FLX treatment after 4 weeks. We observed significant reductions in [^3^H] MK 801 binding in the DCPU, VCPU, and Nac of the MP + FLX group and the DCPU of the MP group. These results are important considering the recent increases in the combined use of MP and SSRIs. Previous research showing altered NMDAR function in neurologic disorders, like Parkinson’s and Alzheimer’s, and dysregulations in reward-processing, such as addiction, increased drug-seeking behavior, and cross-sensitization, further shows the significance of our data. Nevertheless, further research is necessary to investigate the effects of co-administration of oral MP and FLX on NMDAR functioning.

## Supplementary Information

Below is the link to the electronic supplementary material.

Supplementary Material 1

## Data Availability

No datasets were generated or analysed during the current study.

## References

[1] Danielson ML et al (2024) Adhd prevalence among U.S. children and adolescents in 2022: diagnosis, severity, co-occurring disorders, and treatment. J Clin Child Adolesc Psychol 53(3):343–360 38778436 10.1080/15374416.2024.2335625 PMC11334226

[2] MacDonald HJ et al (2024) The dopamine hypothesis for Adhd: an evaluation of evidence accumulated from human studies and animal models. Front Psychiatry 15:1492126 39619336 10.3389/fpsyt.2024.1492126 PMC11604610

[3] Parlatini V et al (2024) From neurons to brain networks, pharmacodynamics of stimulant medication for Adhd. Neurosci Biobehav Rev 164:105841 39098738 10.1016/j.neubiorev.2024.105841

[4] Jaeschke RR, Sujkowska E, Sowa-Kucma M (2021) Methylphenidate for attention-deficit/hyperactivity disorder in adults: a narrative review. Psychopharmacology 238(10):2667–2691 34436651 10.1007/s00213-021-05946-0 PMC8455398

[5] Zito JM et al (2000) Trends in the prescribing of psychotropic medications to preschoolers. JAMA 283(8):1025–1030 10697062 10.1001/jama.283.8.1025

[6] Faraone SV (2018) The pharmacology of amphetamine and methylphenidate: relevance to the neurobiology of attention-deficit/hyperactivity disorder and other psychiatric comorbidities. Neurosci Biobehav Rev 87:255–270 29428394 10.1016/j.neubiorev.2018.02.001 PMC8063758

[7] Chiappini S et al (2024) Methylphenidate abuse and misuse in patients affected with a psychiatric disorder and a substance use disorder: a systematic review. Front Psychiatry 15:1508732 39624511 10.3389/fpsyt.2024.1508732 PMC11609911

[8] Verghese C, Patel P, Abdijadid S (2025) Methylphenidate, in Statpearls. Treasure Island (FL)

[9] Nowak J et al (2024) The Effects of Chronic Psychostimulant Administration on Bone Health: A Review. Biomedicines. 10.3390/biomedicines12081914 39200379 10.3390/biomedicines12081914 PMC11351835

[10] Uddin SMZ et al (2018) Methylphenidate Regulation of Osteoclasts in a Dose- and Sex-Dependent Manner Adversely Affects Skeletal Mechanical Integrity. Sci Rep 8(1):1515 29367750 10.1038/s41598-018-19894-x PMC5784171

[11] Komatsu DE et al (2012) Chronic Exposure to Methylphenidate Impairs Appendicular Bone Quality in Young Rats. Bone 50(6):1214–1222 22465849 10.1016/j.bone.2012.03.011 PMC3352964

[12] Ortiz LM et al (2024) Psychostimulants Prescribed to Children for Adhd Following Distal Radius Fractures Significantly Reduce Bone Density as a Function of Duration. J Pediatr Orthop B 33(4):399–406 37751375 10.1097/BPB.0000000000001125

[13] Chirokikh AA et al (2023) Combined Methylphenidate and Fluoxetine Treatment in Adolescent Rats Significantly Impairs Weight Gain with Minimal Effects on Skeletal Development. Bone 167:116637 36462772 10.1016/j.bone.2022.116637

[14] Ikemoto S (2010) Brain reward circuitry beyond the mesolimbic dopamine system: a neurobiological theory. Neurosci Biobehav Rev 35(2):129–150 20149820 10.1016/j.neubiorev.2010.02.001 PMC2894302

[15] Semaan A, Khan MK (2025) *N*eurobiology of Addiction, in Statpearls. StatPearls Publishing Copyright © 2025, StatPearls Publishing LLC.: Treasure Island (FL)

[16] Richer K et al (2022) Chronic Treatment and Abstinence from Methylphenidate Exposure Dose-Dependently Changes Glucose Metabolism in the Rat Brain. Brain Res 1780:147799 35074404 10.1016/j.brainres.2022.147799

[17] Connor C et al (2022) Abstinence from Chronic Methylphenidate Exposure Modifies Cannabinoid Receptor 1 Levels in the Brain in a Dose-Dependent Manner. Curr Pharm Des 28(4):331–338 33504296 10.2174/1381612827666210127120411

[18] Jalloh K et al (2021) Chronic Oral Methylphenidate Treatment in Adolescent Rats Promotes Dose-Dependent Effects on Nmda Receptor Binding. Life Sci 264:118708 33186568 10.1016/j.lfs.2020.118708

[19] Carias E et al (2018) Chronic Oral Methylphenidate Treatment Increases Microglial Activation in Rats. J Neural Transm (Vienna) 125(12):1867–1875 30238340 10.1007/s00702-018-1931-z PMC6526704

[20] Robison LS et al (2017) Chronic Oral Methylphenidate Treatment Reversibly Increases Striatal Dopamine Transporter and Dopamine Type 1 Receptor Binding in Rats. J Neural Transm (Vienna) 124(5):655–667 28116523 10.1007/s00702-017-1680-4 PMC5400672

[21] Thanos PK et al (2007) Effects of Chronic Oral Methylphenidate on Cocaine Self-Administration and Striatal Dopamine D2 Receptors in Rodents. Pharmacol Biochem Behav 87(4):426–433 17599397 10.1016/j.pbb.2007.05.020

[22] Klein SR et al (2024) Chronic Methylphenidate Effects on Brain Gene Expression: An Exploratory Review. Psychol Res Behav Manag 17:577–592 38379637 10.2147/PRBM.S445719 PMC10876479

[23] Arnavut E et al (2022) Abstinence Following Intermittent Methylphenidate Exposure Dose-Dependently Modifies Brain Glucose Metabolism in the Rat Brain. Synapse 76(9–10):17–30 35730134 10.1002/syn.22243

[24] Senior D et al (2023) Behavioral, neurochemical and developmental effects of chronic oral methylphenidate: a review. J Pers Med 13(4) 10.3390/jpm13040574 PMC10144804 37108960

[25] Kalinowski L et al (2020) Brief and extended abstinence from chronic oral methylphenidate treatment produces reversible behavioral and physiological effects. Dev Psychobiol 62(2):170–180 31456229 10.1002/dev.21902 PMC7028498

[26] Carias E et al (2019) Weekday-Only Chronic Oral Methylphenidate Self-Administration in Male Rats: Reversibility of the Behavioral and Physiological Effects. Behav Brain Res 356:189–196 30149034 10.1016/j.bbr.2018.08.014 PMC6317517

[27] Martin C et al (2018) Recovery from Behavior and Developmental Effects of Chronic Oral Methylphenidate Following an Abstinence Period. Pharmacol Biochem Behav 172:22–32 30030127 10.1016/j.pbb.2018.07.001 PMC6319957

[28] Cheng J et al (2014) Methylphenidate Exerts Dose-Dependent Effects on Glutamate Receptors and Behaviors. Biol Psychiatry 76(12):953–962 24832867 10.1016/j.biopsych.2014.04.003 PMC4194277

[29] Cuffe SP et al (2020) Adhd and Psychiatric Comorbidity: Functional Outcomes in a School-Based Sample of Children. J Atten Disord 24(9):1345–1354 26610741 10.1177/1087054715613437 PMC4879105

[30] Faraone SV et al (2021) The World Federation of Adhd International Consensus Statement: 208 Evidence-Based Conclusions About the Disorder. Neurosci Biobehav Rev 128:789–818 33549739 10.1016/j.neubiorev.2021.01.022 PMC8328933

[31] Gillberg C et al (2004) Co-Existing Disorders in Adhd -- Implications for Diagnosis and Intervention. Eur Child Adolesc Psychiatry 13(Suppl 1):I80-92 15322959 10.1007/s00787-004-1008-4

[32] Biederman J et al (1993) Patterns of Psychiatric Comorbidity, Cognition, and Psychosocial Functioning in Adults with Attention Deficit Hyperactivity Disorder. Am J Psychiatry 150(12):1792–1798 8238632 10.1176/ajp.150.12.1792

[33] Tam J et al (2020) U.S. Simulation of Lifetime Major Depressive Episode Prevalence and Recall Error. Am J Prev Med 59(2):e39–e47 32446751 10.1016/j.amepre.2020.03.021 PMC7375917

[34] Chu A, Wadhwa R (2025) Selective Serotonin Reuptake Inhibitors, in Statpearls. Treasure Island (FL)

[35] Zhou FM et al (2005) Corelease of dopamine and serotonin from striatal dopamine terminals. Neuron 46(1):65–74 15820694 10.1016/j.neuron.2005.02.010

[36] Skolnick P et al (1996) Adaptation of N-Methyl-D-Aspartate (Nmda) Receptors following antidepressant treatment: implications for the pharmacotherapy of depression. Pharmacopsychiatry 29(1):23–26 8852530 10.1055/s-2007-979537

[37] Bhagwagar Z et al (2004) Increased brain gaba concentrations following acute administration of a selective serotonin reuptake inhibitor. Am J Psychiatry 161(2):368–370 14754790 10.1176/appi.ajp.161.2.368

[38] Lazarevic V et al (2019) Fluoxetine Suppresses Glutamate- and Gaba-Mediated Neurotransmission by Altering Snare Complex. Int J Mol Sci. 10.3390/ijms20174247 31480244 10.3390/ijms20174247 PMC6747167

[39] Spurny B et al (2021) Effects of Ssri Treatment on Gaba and Glutamate Levels in an Associative Relearning Paradigm. NeuroImage 232:117913 33657450 10.1016/j.neuroimage.2021.117913 PMC7610796

[40] Van Waes V et al (2010) Selective Serotonin Reuptake Inhibitor Antidepressants Potentiate Methylphenidate (Ritalin)-Induced Gene Regulation in the Adolescent Striatum. Eur J Neurosci 32(3):435–447 20704593 10.1111/j.1460-9568.2010.07294.x PMC2921647

[41] Steiner H, Van Waes V (2013) Addiction-Related Gene Regulation: Risks of Exposure to Cognitive Enhancers Vs. Other Psychostimulants. Prog Neurobiol 100:60–80 23085425 10.1016/j.pneurobio.2012.10.001 PMC3525776

[42] Moon C et al (2021) Fluoxetine potentiates oral methylphenidate-induced gene regulation in the rat striatum. Mol Neurobiol 58(10):4856–4870 34213723 10.1007/s12035-021-02466-y PMC8500935

[43] Robison LS et al (2017) Sex differences in the physiological and behavioral effects of chronic oral methylphenidate treatment in rats. Front Behav Neurosci 11:53 28400722 10.3389/fnbeh.2017.00053 PMC5368228

[44] Thanos PK et al (2023) Combined chronic oral methylphenidate and fluoxetine treatment during adolescence: effects on behavior. Curr Pharm Biotechnol 24(10):1307–1314 36306463 10.2174/1389201024666221028092342

[45] Thanos PK et al (2015) A pharmacokinetic model of oral methylphenidate in the rat and effects on behavior. Pharmacol Biochem Behav 131:143–153 25641666 10.1016/j.pbb.2015.01.005 PMC4461871

[46] Lagamjis G et al (2025) Combined chronic oral methylphenidate and fluoxetine decreases D2r levels in the caudate putamen and nucleus accumbens. Neurochem Res 50(4):230 40643764 10.1007/s11064-025-04481-0 PMC12254183

[47] Lantry AM et al (2025) Combined Chronic Oral Methylphenidate and Fluoxetine Treatment Increases Cb1 Receptor Density in the Somatosensory Forelimb Region. Neuroscience Letters. 10.1016/j.neulet.2025.138407 41046046 10.1016/j.neulet.2025.138407

[48] Kuczenski R, Segal DS (2002) Exposure of adolescent rats to oral methylphenidate: preferential effects on extracellular norepinephrine and absence of sensitization and cross-sensitization to methamphetamine. J Neurosci 22(16):7264–7271 12177221 10.1523/JNEUROSCI.22-16-07264.2002 PMC6757883

[49] Senior D et al (2023) Chronic Oral Methylphenidate Plus Fluoxetine Treatment in Adolescent Rats Increases Cocaine Self-Administration. Addict Neurosci. 10.1016/j.addicn.2023.100127 38274857 10.1016/j.addicn.2023.100127 PMC10809890

[50] Traynelis SF et al (2010) Glutamate Receptor Ion Channels: Structure, Regulation, and Function. Pharmacol Rev 62(3):405–496 20716669 10.1124/pr.109.002451 PMC2964903

[51] Bliss TV, Collingridge GL, Morris RG (2014) Synaptic Plasticity in Health and Disease: Introduction and Overview. Philos Trans R Soc Lond B Biol Sci 369(1633):20130129 24298133 10.1098/rstb.2013.0129 PMC3843863

[52] Volianskis A et al (2015) Long-Term Potentiation and the Role of N-Methyl-D-Aspartate Receptors. Brain Res 1621:5–16 25619552 10.1016/j.brainres.2015.01.016 PMC4563944

[53] Zorumski CF, Izumi Y (2012) Nmda receptors and metaplasticity: mechanisms and possible roles in neuropsychiatric disorders. Neurosci Biobehav Rev 36(3):989–1000 22230702 10.1016/j.neubiorev.2011.12.011 PMC3288588

[54] Petrie RX, Reid IC, Stewart CA (2000) The N-methyl-D-aspartate receptor, synaptic plasticity, and depressive disorder. A critical review. Pharmacol Ther 87(1):11–25 10924739 10.1016/s0163-7258(00)00063-2

[55] Underwood MD et al (2020) Less Nmda receptor binding in dorsolateral prefrontal cortex and anterior cingulate cortex associated with reported early-life adversity but not suicide. Int J Neuropsychopharmacol 23(5):311–318 32060512 10.1093/ijnp/pyaa009 PMC7251634

[56] Abdallah CG et al (2015) Ketamine and rapid-acting antidepressants: a window into a new neurobiology for mood disorder therapeutics. Annu Rev Med 66:509–523 25341010 10.1146/annurev-med-053013-062946 PMC4428310

[57] Stewart CA, Reid IC (2002) Antidepressant mechanisms: functional and molecular correlates of excitatory amino acid neurotransmission. Mol Psychiatry 7(Suppl 1):S15-22 11986991 10.1038/sj.mp.4001014

[58] Crossman AR, Mitchell IJ, Sambrook MA (1985) Regional brain uptake of 2-deoxyglucose in N-methyl-4-phenyl-1,2,3,6-tetrahydropyridine (Mptp)-induced parkinsonism in the macaque monkey. Neuropharmacology 24(6):587–591 3875056 10.1016/0028-3908(85)90070-x

[59] Parameshwaran K, Dhanasekaran M, Suppiramaniam V (2008) Amyloid beta peptides and glutamatergic synaptic dysregulation. Exp Neurol 210(1):7–13 18053990 10.1016/j.expneurol.2007.10.008

[60] Hopf FW (2017) Do specific Nmda receptor subunits act as gateways for addictive behaviors? Genes Brain Behav 16(1):118–138 27706932 10.1111/gbb.12348 PMC5351810

[61] Thanos PK et al (2010) Conditioned place preference and locomotor activity in response to methylphenidate, amphetamine and cocaine in mice lacking dopamine D4 receptors. J Psychopharmacol 24(6):897–904 19282420 10.1177/0269881109102613 PMC2878389

[62] Standaert DG et al (1999) Expression of Nmda glutamate receptor subunit Mrnas in neurochemically identified projection and interneurons in the striatum of the rat. Brain Res Mol Brain Res 64(1):11–23 9889300 10.1016/s0169-328x(98)00293-9

[63] Cepeda C, Van Dongen AM et al (2009) Frontiers in Neuroscience Nmda and Dopamine: Diverse Mechanisms Applied to Interacting Receptor Systems. Biology of the Nmda Receptor. CRC Press/Taylor & Francis, Taylor & Francis Group, LLC.: Boca Raton (FL) (**Copyright © 2009**) 21204414

[64] Mao LM et al (2009) Stability of Surface Nmda Receptors Controls Synaptic and Behavioral Adaptations to Amphetamine. Nat Neurosci 12(5):602–610 19349975 10.1038/nn.2300 PMC2749993

[65] Zhang X et al (2007) Reversal of Cocaine-Induced Behavioral Sensitization and Associated Phosphorylation of the Nr2b and Glur1 Subunits of the Nmda and Ampa Receptors. Neuropsychopharmacology 32(2):377–387 16794574 10.1038/sj.npp.1301101

[66] Stefani MR, Moghaddam B (2005) Transient N-methyl-D-aspartate receptor blockade in early development causes lasting cognitive deficits relevant to schizophrenia. Biol Psychiatry 57(4):433–436 15705361 10.1016/j.biopsych.2004.11.031

[67] Coyle JT et al (2012) Glutamatergic Synaptic Dysregulation in Schizophrenia: Therapeutic Implications. Handb Exp Pharmacol 213:267–95 10.1007/978-3-642-25758-2_10 PMC4817347 23027419

[68] Li JT et al (2016) Repeated blockade of nmda receptors during adolescence impairs reversal learning and disrupts gabaergic interneurons in rat medial prefrontal cortex. Front Mol Neurosci 9:17 26973457 10.3389/fnmol.2016.00017 PMC4776083

[69] Roeser J et al (2025) Chronic combined oral methylphenidate and fluoxetine increases inflammation in somatosensory and mesolimbic brain regions. Neurochem Res 50(6):352 41212420 10.1007/s11064-025-04608-3 PMC12602612

[70] Khan A et al (2024) The Role of Fatty Acid-Binding Protein 5 and 7 on Locomotor, Anxiety and Social Behavior: Interaction with Nmda Signaling. Neurosci Lett 836:137862 38851448 10.1016/j.neulet.2024.137862

[71] Paxinos G, Watson C (1982) The Rat Brain in Stereotaxic Coordinates. Academic Press, Sydney

[72] Selemon LD (2013) A Role for Synaptic Plasticity in the Adolescent Development of Executive Function. Transl Psychiatry 3(3):e238 23462989 10.1038/tp.2013.7 PMC3625918

[73] Isaac JT et al (1997) Silent Synapses during Development of Thalamocortical Inputs. Neuron 18(2):269–280 9052797 10.1016/s0896-6273(00)80267-6

[74] Wiegert JS, Oertner TG (2013) Long-term depression triggers the selective elimination of weakly integrated synapses. Proc Natl Acad Sci U S A 110(47):E4510–E4519 24191047 10.1073/pnas.1315926110 PMC3839749

[75] Sun WL et al (2009) Cocaine effects on dopamine and nmda receptors interactions in the striatum of fischer rats. Brain Res Bull 80(6):377–381 19716863 10.1016/j.brainresbull.2009.08.016 PMC2764833

[76] Chen BT et al (2013) Rescuing Cocaine-Induced Prefrontal Cortex Hypoactivity Prevents Compulsive Cocaine Seeking. Nature 496(7445):359–362 23552889 10.1038/nature12024

[77] McGinty JF, Zelek-Molik A, Sun WL (2015) Cocaine self-administration causes signaling deficits in corticostriatal circuitry that are reversed by Bdnf in early withdrawal. Brain Res 1628(Pt A):82–7 25268928 10.1016/j.brainres.2014.09.050 PMC4377116

[78] Urban KR, Li YC, Gao WJ (2013) Treatment with a clinically-relevant dose of methylphenidate alters Nmda receptor composition and synaptic plasticity in the juvenile rat prefrontal cortex. Neurobiol Learn Mem 101:65–74 23333502 10.1016/j.nlm.2013.01.004 PMC3602399

[79] Kuczenski R, Segal DS (2005) Stimulant actions in rodents: implications for attention-deficit/hyperactivity disorder treatment and potential substance abuse. Biol Psychiatry 57(11):1391–1396 15950013 10.1016/j.biopsych.2004.12.036

[80] Prieto-Gómez B et al (2005) Methylphenidate and amphetamine modulate differently the nmda and ampa glutamatergic transmission of dopaminergic neurons in the ventral tegmental area. Life Sci 77(6):635–649 15921995 10.1016/j.lfs.2004.10.076

[81] Pittenger C, Sanacora G, Krystal JH (2007) The Nmda receptor as a therapeutic target in major depressive disorder. CNS Neurol Disord Drug Targets 6(2):101–115 17430148 10.2174/187152707780363267

[82] Szasz BK et al (2007) Direct Inhibitory Effect of Fluoxetine on N-Methyl-D-Aspartate Receptors in the Central Nervous System. Biol Psychiatry 62(11):1303–1309 17659262 10.1016/j.biopsych.2007.04.014

[83] Kiss JP et al (2012) Glun2b-Containing Nmda Receptors as Possible Targets for the Neuroprotective and Antidepressant Effects of Fluoxetine. Neurochem Int 60(2):170–176 22197911 10.1016/j.neuint.2011.12.005

[84] Yuen EY et al (2005) Serotonin 5-Ht1a Receptors Regulate Nmda Receptor Channels through a Microtubule-Dependent Mechanism. J Neurosci 25(23):5488–5501 15944377 10.1523/JNEUROSCI.1187-05.2005 PMC6724987

[85] Hrabak M et al (2025) Correction: Vilazodone, a Novel Ssri Antidepressant with 5-Ht1a Partial Agonist Properties: Diminished Potentiation of Chronic Oral Methylphenidate-Induced Dynorphin Expression in the Striatum in Adolescent Male Rats. Mol Neurobiol 62(4):4533 39541073 10.1007/s12035-024-04621-7

[86] Yager LM et al (2015) The Ins and Outs of the Striatum: Role in Drug Addiction. Neuroscience 301:529–541 26116518 10.1016/j.neuroscience.2015.06.033 PMC4523218

[87] Graybiel AM, Grafton ST (2015) The Striatum: Where Skills and Habits Meet. Cold Spring Harb Perspect Biol 7(8):a021691 26238359 10.1101/cshperspect.a021691 PMC4526748

[88] Nambu A (2004) A new dynamic model of the cortico-basal ganglia loop. Prog Brain Res 143:461–466 14653188 10.1016/S0079-6123(03)43043-4

[89] Wise RA (2009) Roles for nigrostriatal–not just mesocorticolimbic–dopamine in reward and addiction. Trends Neurosci 32(10):517–524 19758714 10.1016/j.tins.2009.06.004 PMC2755633

[90] Toy WA et al (2014) Treadmill Exercise Reverses Dendritic Spine Loss in Direct and Indirect Striatal Medium Spiny Neurons in the 1-Methyl-4-Phenyl-1,2,3,6-Tetrahydropyridine (Mptp) Mouse Model of Parkinson’s Disease. Neurobiol Dis 63:201–209 24316165 10.1016/j.nbd.2013.11.017 PMC3940446

[91] Lima MM, Reksidler AB, Vital MA (2009) The Neurobiology of the Substantia Nigra Pars Compacta: From Motor to Sleep Regulation. J Neural Transm Suppl 73:135–45 10.1007/978-3-211-92660-4_11 20411774

[92] Pan HS, Penney JB, Young AB (1985) Gamma-aminobutyric acid and benzodiazepine receptor changes induced by unilateral 6-hydroxydopamine lesions of the medial forebrain bundle. J Neurochem 45(5):1396–1404 2995585 10.1111/j.1471-4159.1985.tb07205.x

[93] Macdonald PA, Monchi O (2011) Differential effects of dopaminergic therapies on dorsal and ventral striatum in Parkinson’s disease: implications for cognitive function. Parkinsons Dis 2011:572743 21437185 10.4061/2011/572743 PMC3062097

[94] Zhang K et al (2025) Nmda receptors in neurodegenerative diseases: mechanisms and emerging therapeutic strategies. Front Aging Neurosci 17:1604378 40778304 10.3389/fnagi.2025.1604378 PMC12328396

[95] Ułas J et al (1994) Selective increase of nmda-sensitive glutamate binding in the striatum of Parkinson’s disease, alzheimer’s disease, and mixed Parkinson’s Disease/Alzheimer’s disease patients: an autoradiographic study. J Neurosci 14(11 Pt 1):6317–6324 7965038 10.1523/JNEUROSCI.14-11-06317.1994 PMC6577237

[96] Hallett PJ, Standaert DG (2004) Rationale for and use of NMDA receptor antagonists in Parkinson’s disease. Pharmacol Ther 102(2):155–174 15163596 10.1016/j.pharmthera.2004.04.001

[97] Shi J, Townsend M, Constantine-Paton M (2000) Activity-dependent induction of tonic calcineurin activity mediates a rapid developmental downregulation of NMDA receptor currents. Neuron 28(1):103–114 11086987 10.1016/s0896-6273(00)00089-1

[98] Oster Y, Schramm M (1993) Down-regulation of NMDA receptor activity by NMDA. Neurosci Lett 163(1):85–88 8295741 10.1016/0304-3940(93)90235-d

[99] Devan BD, McDonald RJ, White NM (1999) Effects of medial and lateral caudate-putamen lesions on place- and cue-guided behaviors in the water maze: relation to thigmotaxis. Behav Brain Res 100(1–2):5–14 10212049 10.1016/s0166-4328(98)00107-7

[100] Yin HH, Knowlton BJ, Balleine BW (2004) Lesions of dorsolateral striatum preserve outcome expectancy but disrupt habit formation in instrumental learning. Eur J Neurosci 19(1):181–189 14750976 10.1111/j.1460-9568.2004.03095.x

[101] Goodman J, Leong KC, Packard MG (2023) Nmda Receptor Blockade in the Dorsolateral Striatum Impairs Consolidation but Not Retrieval of Habit Memory. Neurobiol Learn Mem 197:107709 36503101 10.1016/j.nlm.2022.107709

[102] Yin HH et al (2005) The Role of the Dorsomedial Striatum in Instrumental Conditioning. Eur J Neurosci 22(2):513–523 16045504 10.1111/j.1460-9568.2005.04218.x

[103] Huang H et al (2017) Mapping Dorsal and Ventral Caudate in Older Adults: Method and Validation. Front Aging Neurosci 9:91 28420985 10.3389/fnagi.2017.00091 PMC5378713

[104] Di Martino A et al (2008) Functional Connectivity of Human Striatum: A Resting State Fmri Study. Cereb Cortex 18(12):2735–2747 18400794 10.1093/cercor/bhn041

[105] Martinez D et al (2003) Imaging Human Mesolimbic Dopamine Transmission with Positron Emission Tomography. Part Ii: Amphetamine-Induced Dopamine Release in the Functional Subdivisions of the Striatum. J Cereb Blood Flow Metab 23(3):285–300 12621304 10.1097/01.WCB.0000048520.34839.1A

[106] Vonsattel JP (2008) Huntington disease models and human neuropathology: similarities and differences. Acta Neuropathol 115(1):55–69 17978822 10.1007/s00401-007-0306-6 PMC2847401

[107] Haynos AF et al (2021) Resting State Hypoconnectivity of Reward Networks in Binge Eating Disorder. Cereb Cortex 31(5):2494–2504 33415334 10.1093/cercor/bhaa369 PMC8248831

[108] Haynos AF et al (2019) Resting State Functional Connectivity of Networks Associated with Reward and Habit in Anorexia Nervosa. Hum Brain Mapp 40(2):652–662 30251758 10.1002/hbm.24402 PMC6314844

[109] Ong D et al (2012) Size and Shape of the Caudate Nucleus in Individuals with Bipolar Affective Disorder. Aust N Z J Psychiatry 46(4):340–351 22368240 10.1177/0004867412440191 PMC3328643

[110] Aouizerate B et al (2004) Deep brain stimulation of the ventral caudate nucleus in the treatment of obsessive-compulsive disorder and major depression. Case report. J Neurosurg 101(4):682–686 15481726 10.3171/jns.2004.101.4.0682

[111] Jastrzębska K et al (2016) Loss of Nmda Receptors in Dopamine Neurons Leads to the Development of Affective Disorder-Like Symptoms in Mice. Sci Rep 6:37171 27853270 10.1038/srep37171 PMC5112557

[112] Vibholm AK et al (2020) Activation of Nmda Receptor Ion Channels by Deep Brain Stimulation in the Pig Visualised with [(18)F]Ge-179 Pet. Brain Stimul 13(4):1071–1078 32388196 10.1016/j.brs.2020.03.019

[113] Świetlik D (2024) Deep Brain Stimulation Combined with Nmda Antagonist Therapy in the Treatment of Alzheimer’s Disease: In Silico Trials. J Clin Med. 10.3390/jcm13247759 39768683 10.3390/jcm13247759 PMC11728097

[114] Giacobbe P, Kennedy SH (2006) Deep brain stimulation for treatment-resistant depression: a psychiatric perspective. Curr Psychiatry Rep 8(6):437–444 17094923 10.1007/s11920-006-0048-5

[115] Ferré S et al (2010) Adenosine-Cannabinoid Receptor Interactions. Implications for Striatal Function. Br J Pharmacol 160(3):443–453 20590556 10.1111/j.1476-5381.2010.00723.x PMC2931547

[116] Ubeda-Bañon I et al (2007) Projections from the Posterolateral Olfactory Amygdala to the Ventral Striatum: Neural Basis for Reinforcing Properties of Chemical Stimuli. BMC Neurosci 8:103 18047654 10.1186/1471-2202-8-103 PMC2216080

[117] Morales M, Margolis EB (2017) Ventral tegmental area: cellular heterogeneity, connectivity and behaviour. Nat Rev Neurosci 18(2):73–85 28053327 10.1038/nrn.2016.165

[118] Hanlon CA, Dowdle LT, Jones JL (2016) Biomarkers for success: using neuroimaging to predict relapse and develop brain stimulation treatments for cocaine-dependent individuals. Int Rev Neurobiol 129:125–156 27503451 10.1016/bs.irn.2016.06.006 PMC5492974

[119] Cai J, Tong Q (2022) Anatomy and Function of Ventral Tegmental Area Glutamate Neurons. Front Neural Circuits 16:867053 35669454 10.3389/fncir.2022.867053 PMC9164627

[120] Saddoris MP et al (2015) Differential Dopamine Release Dynamics in the Nucleus Accumbens Core and Shell Reveal Complementary Signals for Error Prediction and Incentive Motivation. J Neurosci 35(33):11572–11582 26290234 10.1523/JNEUROSCI.2344-15.2015 PMC4540796

[121] Blum K et al (2012) Sex, drugs, and rock ‘N’ roll: hypothesizing common mesolimbic activation as a function of reward gene polymorphisms. J Psychoact Drugs 44(1):38–55 10.1080/02791072.2012.662112 PMC4040958 22641964

[122] Valencia Garcia S, Fort P (2018) Nucleus accumbens, a new sleep-regulating area through the integration of motivational stimuli. Acta Pharmacol Sin 39(2):165–166 29283174 10.1038/aps.2017.168 PMC5800466

[123] Hansen KB et al (2017) Nmda Receptors in the Central Nervous System. Methods Mol Biol 1677:1–80 28986865 10.1007/978-1-4939-7321-7_1 PMC7325486

[124] Jewett BE, Thapa B (2025) Physiology, Nmda Receptor, in Statpearls. StatPearls Publishing Copyright © 2025, StatPearls Publishing LLC.: Treasure Island (FL)

[125] Joffe ME, Vitter SR, Grueter BA (2017) Glun1 Deletions in D1- and A2a-Expressing Cell Types Reveal Distinct Modes of Behavioral Regulation. Neuropharmacology 112Pt A:172–180 10.1016/j.neuropharm.2016.03.026 PMC5031515 27012890

