# Supplementary material for: Chronic Co-administration of Methylphenidate and Fluoxetine Reduces Striatal NMDA Receptor Binding in Adolescent Rats
Source: Neurochem Res. 2026 Apr 3;51(2):130. doi: 10.1007/s11064-026-04743-5 (PMC13048928; doi:10.1007/s11064-026-04743-5)
Supplement: Supplementary file 1 — Supplementary Material 1 [file 11064_2026_4743_MOESM1_ESM.docx]

**Supplemental Table 1**

| Region of Interest | F Value (DFn, DFd) | P-Values |
| --- | --- | --- |
| Amygdala (Amyg) | F (3,28) = 1.389 | 0.2666 |
| Auditory Cortex (AuD) | F (3,28) = 0.2873 | 0.8341 |
| Cingulate Gyrus (Cg) | F (3, 28) = 0.5732 | 0.6373 |
| Dorsal CPU (D CPU) | F (3, 28) = 1.541 | 0.2259 |
| Dorsolateral CPU (DLCPU) | F (3,28) = 1.696 | 0.1905 |
| Dorsomedial CPU (DMCPU) | F (3,28) = 1.596 | 0.2125 |
| Ectorhinal Cortex (Ect) | F (3, 28) = 0.1936 | 0.8999 |
| Entorhinal Cortex (Ent) | F (3, 28) = 0.4238 | 0.7374 |
| Hippocampus (HP) | F (3, 28) = 0.07728 | 0.9718 |
| Hypothalamus (Hyp) | F (3, 21) = 1.150 | 0.3520 |
| Infralimbic (IL) | F (3, 28) = 0.6717 | 0.5766 |
| Insular Cortex (INS) | F (3, 28) = 0.4043 | 0.7511 |
| Primary Motor (M1) | F (3, 28) = 0.4511 | 0.7185 |
| Secondary Motor (M2) | F (3, 28) = 0.5166 | 0.6743 |
| Nucleus Accumbens Core (Nac Core) | F (3, 27) = 1.426 | 0.2568 |
| Nucleus Accumbens Shell (Nac Shell) | F (3, 26) = 1.599 | 0.2136 |
| Piriform Cortex (Piri) | F (3, 28) = 0.3599 | 0.7824 |
| Perirhinal Cortex (PRh) | F (3, 28) = 0.2685 | 0.8475 |
| Prelimbic Cortex (PrL) | F (3, 28) = 0.6787 | 0.5724 |
| Retrosplenial Cortex (RS) | F (3, 28) = 0.5744 | 0.6366 |
| Sensory Barrel Field (S(BF)) | F (3, 28) = 0.1727 | 0.9140 |
| Sensory Forelimb (S(FL)) | F (3, 28) = 0.6927 | 0.5642 |
| Sensory Hindlimb (S(HL)) | F (3, 28) = 0.3499 | 0.7895 |
| Sensory Jaw (S(Jaw)) | F (3, 28) = 1.014 | 0.4011 |
| Sensory Trunk (S(Tr)) | F (3, 26) = 0.3589 | 0.7832 |
| Sensory Upper lip S(ULP) | F (3, 28) = 0.5439 | 0.6563 |
| Primary Sensory Area (S1) | F (3, 27) = 0.3815 | 0.7671 |
| Secondary Sensory Area (S2) | F (3, 27) = 0.6627 | 0.5822 |
| Thalamus (Th) | F (3, 28) = 0.6940 | 0.5634 |
| Visual Cortex (Vis) | F (3, 28) = 0.2032 | 0.8933 |
| Ventral CPU (VCPU) | F (3, 28) = 1.1753 | 0.1790 |
| Ventrolateral CPU (VLCPU) | F (3, 28) = 1.418 | 0.2584 |
| Ventromedial CPU (VMCPU) | F (2, 28) = 1.358 | 0.2759 |

**Supplemental Table 1.** Table detailing all ROIs that showed no significance between all treatment groups using one-way ANOVA analysis. P > 0.05 for all ROIs. DFn indicates degrees of freedom for numerator. DFd indicates degrees of freedom for denominator.
